# Report of the Treasury Cattle Commission on the Lung Plague of Cattle, or Contagious Pleuro-Pneumonia

**Published:** 1882-10

**Authors:** 


### REVIEWS.

---

**Report of the Treasury Cattle Commission on the Lung Plague of Cattle, or Contagious Pleuro Pneumonia.** Pp. 138, 8vo. Washington: 1882.

This Report is an ably-written document, and does full justice to the important subject which it treats. Adopting by preference the term Lung Plague for the disease as indicating its specific character, the author gives a concise history of it from the time of Aristotle to the present day. It tells how the infection, at first confined to Central and Western Europe, slowly travelled eastward and southward, soon reaching the British Isles, and finally, our country, through the port of New York, from which point it travelled only southward, owing to the opposing current of cattle traffic; and how it found its way into Massachusetts by direct importation, but was immediately stamped out—thanks to the prompt and efficient action of the authorities. It is proved, also, that it arises and spreads invariably by direct contagion, and is peculiar to the bovine genus; shows the inefficacy of inoculation, its great expense, and the impossibility or making it universal, advising the better method of extinction; shows how faulty the present system of quarantine is, and suggests an improved one.

We are glad to know that the Treasury Department has recognized the importance of the subject of Professor Law's Report and is about to establish quarantine stations for imported cattle at New York and other seaports, under the authority of an appropriation of $50,000 by Congress. But we sincerely hope that the department will not stop here, but promptly follow it up by undertaking and putting in execution the plan of extinction or "stamping out" as the most efficient and, in the end, the most economical one of thoroughly exterminating this terrible disease from the country.

